# Synthesis of Silver–Calcium Phosphate Visible Light Responsive Photocatalytic Materials and Their Antibacterial Properties

**DOI:** 10.3390/ma18204789

**Published:** 2025-10-20

**Authors:** Hiroaki Onoda, Chihiro Izaki

**Affiliations:** 1 Department of Biomolecular Chemistry, Faculty of Science and Technology, Kyoto Prefectural University, 1-5, Shimogamo Nakaragi-cyo, Sakyo-ku, Kyoto 606-8522, Japan; 2 Department of Informatics and Environmental Sciences, Faculty of Life and Environmental Sciences, Kyoto Prefectural University, 1-5, Shimogamo Nakaragi-cyo, Sakyo-ku, Kyoto 606-8522, Japan; st11025653@gmail.com

**Keywords:** visible light photocatalytic activity, hydrothermal process, inorganic phosphate, antimicrobial activity

## Abstract

Photocatalytic materials use light energy to decompose harmful substances, antifouling, and deodorize. However, photocatalytic materials currently in practical use utilize ultraviolet light, and considering the ratio of ultraviolet light in sunlight and indoor specifications, there is a need for photocatalysts that can act with visible light. Silver phosphate is a photocatalytic material that works with visible light, but the silver ions make it expensive and difficult to put to practical use. This material is also expected to have antibacterial properties derived from silver ions. In this study, we prepared silver–calcium phosphate with a reduced amount of silver. The composition, photocatalytic activity, and antibacterial properties of the obtained samples were evaluated to examine the potential of the novel material.

## 1. Introduction

Photocatalysis is a phenomenon that uses light energy to break down harmful chemicals and other substances [1,2,3,4]. This effect is very useful because it works at room temperature and pressure. Currently, this effect is widely used in water treatment, air purification, and antimicrobial coatings [5,6,7,8,9,10]. Typical compounds used as photocatalysts are titanium dioxide and zinc oxide [11,12,13,14]. These work under ultraviolet light and hardly work indoors, where ultraviolet light is scarce. Its efficiency is also poor when used outdoors, since only about 6% of sunlight is ultraviolet light [15].

Visible light, on the other hand, is present in about 52% of sunlight, much more than ultraviolet light. Visible light is also supplied indoors by fluorescent lamps and LEDs. Therefore, photocatalytic materials are expected to work more effectively if visible light photocatalysts can be utilized as an energy source. In light of the above, there is a need for photocatalytic materials that respond to visible light photocatalysts. One such visible light photocatalyst is silver phosphate [16,17,18,19]. However, silver phosphate has the disadvantage of being a silver compound, which is expensive. Previous studies have developed visible light-responsive photocatalysts with reduced silver usage [20,21]. The photocatalytic activity of silver-zinc phosphate remains unchanged after three reuses, confirming its potential as a promising material.

Silver compounds are also expected to have antimicrobial properties [22,23,24,25]. The antimicrobial properties of silver are demonstrated when silver ions bind to bacterial cell membranes and inhibit protein function or produce reactive oxygen species that kill microorganisms. It is effective against a wide range of bacteria and viruses, and is also effective against drug-resistant bacteria. If the concentration is optimized, it is safe for the human body and can be used in many fields, including medicine, hygiene products, and clothing.

Referencing a prior silver-zinc phosphate study [21], we synthesized phosphates by partially substituting silver with copper, cobalt, calcium, iron (III), and aluminum. Among these, this study reports on silver–calcium phosphate, which showed the highest potential for reducing silver usage with high performance. The composition, photocatalytic activity, and antibacterial properties of the resulting samples were then investigated.

## 2. Materials and Methods

### 2.1. Sample Preparation

Silver nitrate and calcium nitrate were mixed in molar ratios of Ag/Ca = 10/90, 25/75, 30/70, 40/60, and added to 12 mL of 0.5 mol/L phosphoric acid at a molar ratio of (Ag + 2Ca)/P = 3/1. This molar ratio of (Ag + 2Ca)/P = 3/1 is based on that silver is a monovalent cation, calcium is a divalent cation, and phosphoric acid is a trivalent anion. The mixed aqueous solution was subjected to hydrothermal treatment at 160 °C for 20 h. The solutions were then adjusted to pH 5, 7, and 9 with 8 mol/L sodium hydroxide solution. Samples without pH adjustment were also prepared, but the pH was near 1 at that time. By adding 50 mL of acetone, a precipitate was formed, then filtered and dried. Samples were also prepared at hydrothermal treatment temperatures of 120 °C, 140 °C, and 180 °C. In addition, silver phosphate without calcium substitution was also prepared for comparison.

### 2.2. Hue and Composition of Samples

The hue of the samples was evaluated using the L*a*b* color system. TES135 plus color analyzer (TES Electrical Electronic Corp., Taipei, Taiwan) provided the L*a*b* values. The L* values represent whiteness, with 100 indicating white and 0 indicating black. The a* values represent redness, with positive values corresponding to red and negative values to green. On the other hand, the b* values represent yellowness, with positive values corresponding to yellow and negative values to blue.

The crystal structure and chemical bonding of these materials were analyzed using X-ray diffraction (XRD) patterns and infrared (IR) spectra, respectively. XRD patterns were recorded on an X-ray diffractometer (MiniFlex, Rigaku Corp., Akishima, Japan) using monochromatic CuKα radiation (30 kV, 15 mA, 3°/min, step size: 0.02°). The IR spectra of the samples were recorded by the KBr disk method (Resolution: 4 cm^−1^, 16 times scanned) using a HORIBA Fourier-transform infrared (FT-IR) 720 (HORIBA Corp., Kyoto, Japan).

Scanning Electron Microscope (SEM) images of the samples were recorded using a MiniscopeR TM3030Plus (Hitachi High-Tech Corp., Tokyo, Japan) with an accelerating voltage of 5 kV/15 kV.

### 2.3. Photocatalytic and Antibacterial Properties of Samples

The photocatalytic activity of the samples was estimated with the decomposition of methylene blue by LED light (520 lumens, 5.5 W, DS-LN42BG-W, OHM Electric Inc., Tokyo, Japan). The 0.01 g of sample was placed in 4 mL of methylene blue solution (1.0 × 10^−5^ mol/L), and then this solution was irradiated. The decrease in the absorption at about 660 nm was estimated for 120 min.

*E. coli* (Dh5α) was used to evaluate the antibacterial activity of the prepared samples [26,27]. 25 μL of LB broth medium containing *E. coli* was applied to an agar plate, and 30 mg of the pelleted sample was placed on the plate at equal intervals and incubated for 24 h under dark conditions. If the sample has antimicrobial activity, the culture of *E. coli* is inhibited, and a circle called the inhibition circle, in which *E. coli* is not present, is observed. The antimicrobial activity was evaluated by comparing the presence or absence or size of the inhibition circle.

## 3. Results and Discussion

Table 1 shows yields and L*a*b* values. Silver phosphate and calcium phosphate were also evaluated for hue, since they are yellow and white powders, respectively. The sample without pH adjustment showed a decrease in yield as the ratio of silver increased. On the other hand, the samples with Ag/Ca = 25/75 and pH adjustment had yields above 100%. This was thought to be due to the sample’s hydroxide content, which exceeded 100%. When looking at the hue of the samples, the L* values were all above 85, and the a* values were close to zero. On the other hand, the b* value became higher and more yellow as the silver ratio increased. The change in b* value with a change in pH is smaller than the change in Ag/Ca ratio, suggesting that the effect of pH on the silver fraction in the samples is smaller than that of the Ag/Ca ratio. The samples prepared in this study have lower b* values than silver phosphate, which is thought to be due to the effect of calcium salts.

**Figure 1:**
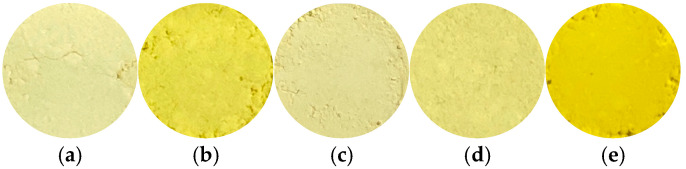
Photographs of samples prepared at various pH values (Ag/Ca = 25/75), (**a**) pH 1, (**b**) 5, (**c**) 7, (**d**) 9, (**e**) Ag_3_PO_4_.

Figure 1 shows photographs of samples prepared at various pH values. Silver phosphate was a strong yellow powder (Figure 1e). The samples prepared in this study exhibited a color between yellow and white, as they were a mixture of silver phosphate and white calcium phosphate. At pH 5 or above, both silver phosphate and calcium phosphate were expected to precipitate completely, eliminating color changes. However, this study showed color changes that were not proportional to pH. The cause of this change is unknown, but it could be said that the color change was smaller compared to pure silver phosphate.

**Figure 2:**
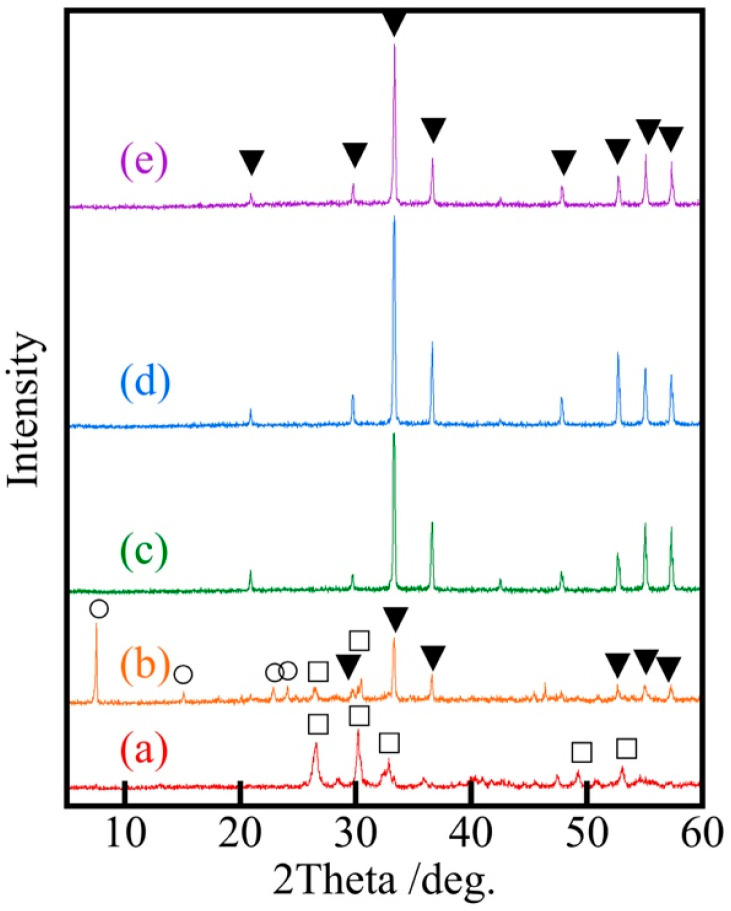
XRD patterns of samples prepared at various Ag ratios, Ag/Ca = x/(100 − x), (a) 10, (b) 25, (c) 30, (d) 40, (e) Ag_3_PO_4_, ▼: AgPO_4_, □: CaHPO_4_, ○: Ca(H_2_PO_4_)_2_·H_2_O.

Figure 2 shows the XRD patterns of samples prepared at various Ag/Ca ratios. Samples with lower silver ratios showed the peaks derived from calcium phosphate, while samples with silver ratios higher than Ag/Ca = 30/70 showed only silver phosphate peaks. The Ag_3_PO_4_ peaks in these samples with a high ratio of silver were as intense as those of silver phosphate without calcium. Silver phosphate was considered to crystallize more easily than calcium phosphate.

**Figure 3:**
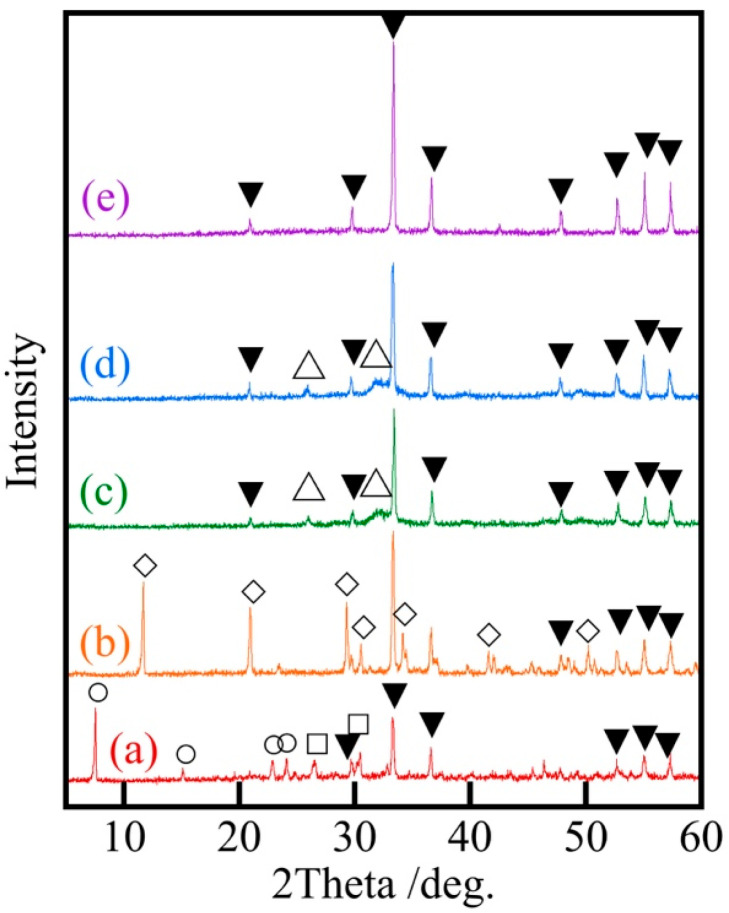
XRD patterns of samples prepared at various pH values (Ag/Ca = 25/75), (a) pH 1, (b) 5, (c) 7, (d) 9, (e) Ag_3_PO_4_, ▼: AgPO_4_, □: CaHPO_4_, ○: Ca(H_2_PO_4_)_2_·H_2_O, ◇: CaHPO_4_·2H_2_O, △: Ca_10_(PO_4_)_6_(OH)_2_.

Figure 3 shows XRD patterns of Ag/Ca = 25/75 samples prepared at various pH levels. The calcium phosphate produced changed from CaHPO_4_ and Ca(H_2_PO_4_)_2_·H_2_O to Ca_10_(PO_4_)_6_(OH)_2_ with increasing pH. This change corresponded to the effect of pH in the preparation of calcium phosphate. Although calcium phosphate is less crystallizable than silver phosphate, peaks of calcium phosphate were observed when Ag/Ca = 25/75.

**Figure 4:**
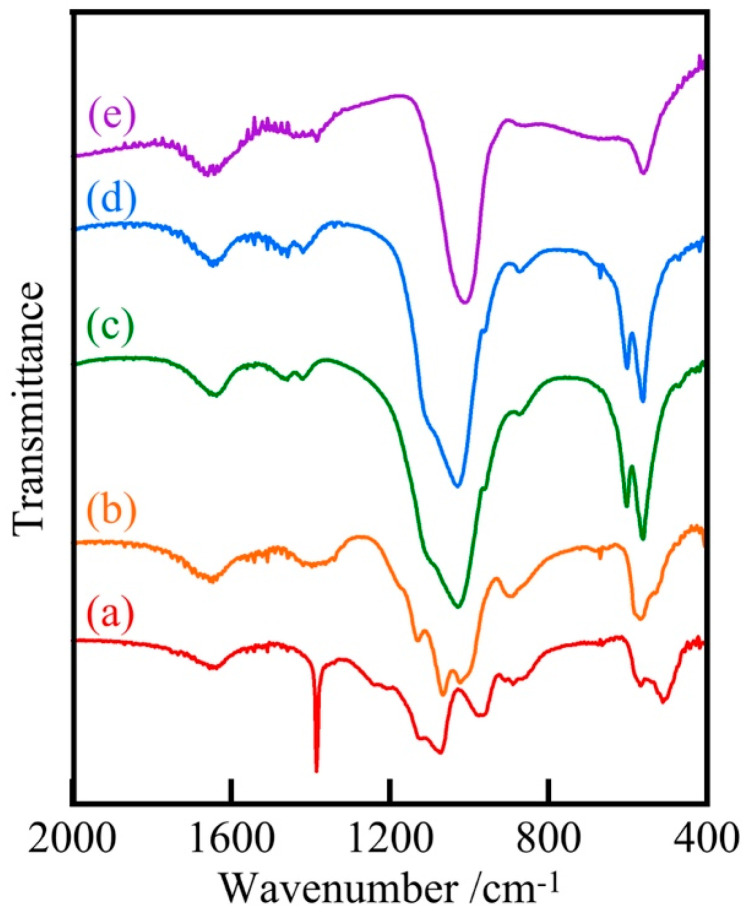
IR spectra of samples prepared at various pH values (Ag/Ca = 25/75), (a) pH 1, (b) 5, (c) 7, (d) 9, (e) Ag_3_PO_4_.

Figure 4 shows the IR spectra of Ag/Ca = 25/75 samples prepared at various pH levels. Many peaks at 510, 570, 960, 1070, 1130 cm^−1^ were observed in samples prepared at pH 5 and below, with a strong peak near 1380 cm^−1^ due to nitrate, and other peaks due to phosphate [28,29]. Many peaks suggested the presence of diverse phosphates. On the other hand, the samples prepared at pH 7 and 9 had fewer peaks. The peaks at 560 and 1030 cm^−1^ in the IR spectra of these samples were similar to those caused by silver phosphate, suggesting that silver phosphate is relatively more likely to produce peaks.

**Figure 5:**
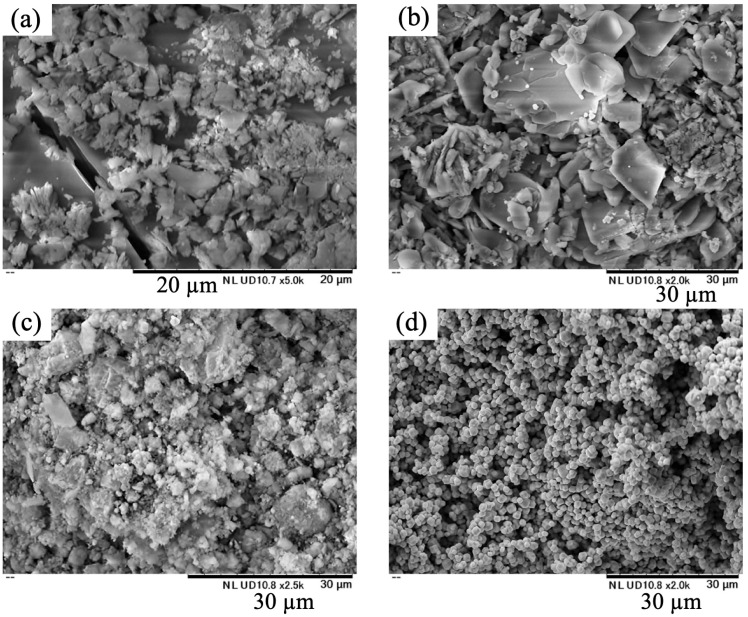
SEM images of samples prepared at various pH values (Ag/Ca = 25/75), (**a**) pH 1, (**b**) 5, (**c**) 7, (**d**) Ag_3_PO_4_.

Figure 5 shows SEM images of Ag/Ca = 25/75 samples prepared at various pH levels. The silver phosphate particles are small, whereas the samples prepared in this study have relatively large particles and flat surfaces. The particles were considered to be larger due to the formation of calcium phosphate.

**Figure 6:**
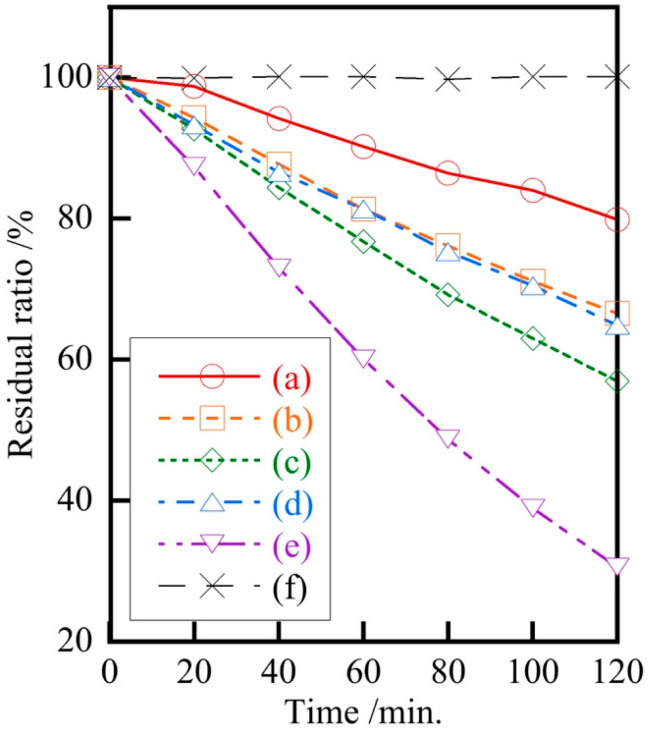
Photocatalytic activity of samples prepared at various pH values (Ag/Ca = 25/75), (a) pH 1, (b) 5, (c) 7, (d) 9, (e) Ag_3_PO_4_, (f) Blank.

Figure 6 shows the photocatalytic activity of Ag/Ca = 25/75 samples prepared at various pH levels. The samples prepared in this study showed visible light photocatalytic activity, although not as good as Ag_3_PO_4_. In a previous study where part of the silver was replaced with zinc [21], the photocatalytic activity increased compared to pure silver phosphate due to a smaller particle size; however, no such phenomenon was observed in this study. It is important to note here that Ag/Ca = 25/75, and despite the much lower amount of silver used, the photocatalytic activity was sufficient. The fact that the photocatalytic activity is still present even when the silver content is reduced shows great potential for the future development of visible light photocatalyst materials.

**Figure 7:**
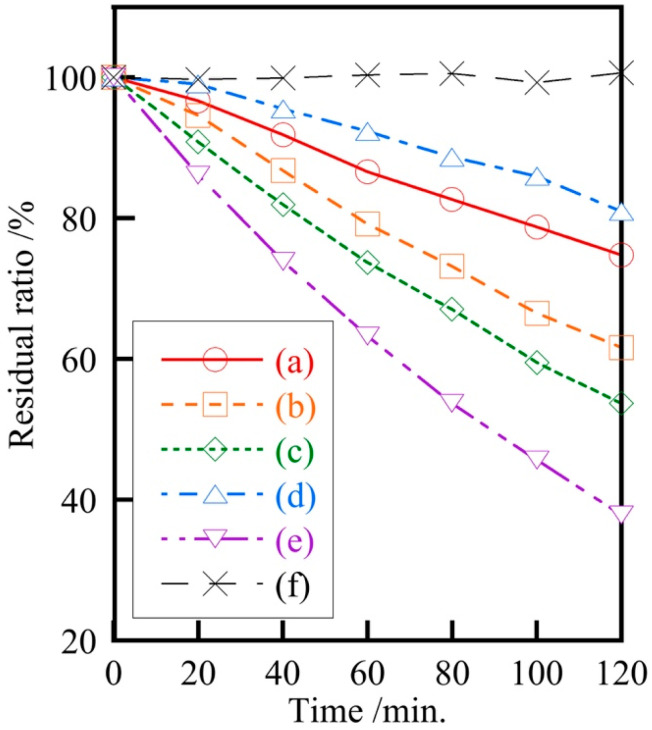
Photocatalytic activity of samples prepared at various temperatures (Ag/Ca = 25/75), (a) 120 °C, (b) 140 °C, (c) 160 °C, (d) 180 °C, (e) Ag_3_PO_4_, (f) Blank.

Figure 7 shows the photocatalytic activity of samples prepared at various hydrothermal treatment temperatures. Although there was no regularity with hydrothermal treatment temperature, the samples prepared at 160 °C had relatively high photocatalytic activity. The sample prepared at 180 °C exhibited reduced decomposition efficiency due to aggregation in the methylene blue solution. The significant change in the photocatalytic activity of the samples by varying the hydrothermal treatment temperature had the potential to be changed by other conditions, suggesting the possibility of obtaining even higher photocatalytic activity in the future.

**Figure 8:**
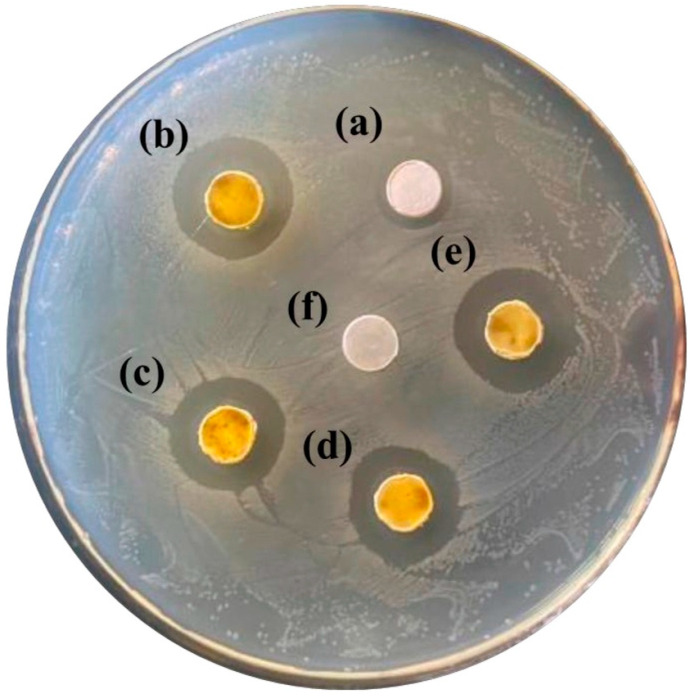
Antimicrobial activity of samples prepared at various Ag ratios, Ag/Ca = x/(100 − x), (a) 10, (b) 25, (c) 30, (d) 40, (e) Ag_3_PO_4_, (f) blank.

Figure 8 shows the antimicrobial properties of samples prepared with various Ag/Ca ratios: the sample with Ag/Ca = 10/90 had a small inhibition circle, indicating that it had slight antimicrobial activity. The other samples had the same level of inhibition circle as that of Ag_3_PO_4_, indicating that the samples showed high antimicrobial activity despite the reduction of silver. This demonstrates that materials with sufficient antimicrobial properties can be produced using less silver.

## 4. Conclusions

Phosphate photocatalytic materials were prepared by replacing some of the silver with calcium in order to control the amount of silver used. Although the substitution of calcium resulted in lower yields, samples could be obtained in higher yields by adjusting the pH. For samples with a higher percentage of silver than Ag/Ca = 30/70, silver phosphate peaks were observed in the XRD pattern, but no calcium phosphate peaks were observed. The Ag/Ca = 25/75 sample showed XRD peaks of both silver and calcium phosphate at all pH levels. The samples in this study were larger particles than silver phosphate. The samples prepared in this study exhibited relatively high visible light photocatalyst activity, despite a sufficiently low proportion of silver. Using *E. coli*, the Ag/Ca = 25/75 sample exhibited antimicrobial activity comparable to that of Ag_3_PO_4_. The fact that the phosphate samples showed sufficiently high photocatalytic activity and antimicrobial activity even with a low ratio of silver indicates the potential for novel materials in the future.

## Figures and Tables

**Table 1:** Yields and L*a*b* values of samples prepared at various Ag ratios, Ag/Ca = x/(100 − x), and various pH values.

x	pH	Yield/%	L*	a*	b*
10	1	50.6	93.67	−0.38	4.56
25	1	38.6	89.94	1.54	24.13
30	1	15.8	94.95	0.33	46.05
40	1	22.5	93.46	1.55	50.12
25	5	129	85.38	−0.46	37.27
25	7	107	89.55	0.43	22.47
25	9	110	85.55	−1.17	31.44
Ag_3_PO_4_	9	-	86.83	0.79	70.16

## Data Availability

The raw data supporting the conclusions of this article will be made available by the authors on request.
